# Urgent air transfers for acute respiratory infections among children from Northern Canada, 2005–2014

**DOI:** 10.1371/journal.pone.0272154

**Published:** 2022-07-28

**Authors:** Caitlin Prendergast, Joan Robinson, Chelsea Caya, Maria E. Perez Trejo, Iline Guan, Veronica Hébert-Murakami, Justina Marianayagam, Zing-Wae Wong, Celia Walker, David M. Goldfarb, Nick Barrowman, Radha Jetty, Joanne Embree, Jesse Papenburg

**Affiliations:** 1 Department of Pediatrics, University of Ottawa, Ottawa, Canada; 2 Department of Pediatrics, University of Alberta, Edmonton, Canada; 3 Research Institute of the McGill University Health Centre, Montreal, Canada; 4 Research Institute of the Children’s Hospital of Eastern Ontario, Ottawa, Canada; 5 Division of Pediatric Infectious Diseases, Department of Pediatrics, Montreal Children’s Hospital, Montreal, Canada; 6 Northern Ontario School of Medicine, Thunder Bay, Canada; 7 Department of Pathology and Laboratory Medicine, University of British Columbia, Vancouver, Canada; 8 Department of Pediatrics, University of Manitoba, Winnipeg, Canada; 9 Division of Microbiology, Department of Clinical Laboratory Medicine, Optilab Montreal, McGill University Health Centre, Montreal, Canada; Public Library of Science, UNITED KINGDOM

## Abstract

**Background:**

The incidence of hospitalizations for acute respiratory infections (ARI) among young Indigenous children from Northern Canada is consistently high. ARIs requiring urgent air transfer can be life-threatening and costly. We aimed to describe their epidemiology, estimate age-specific incidences, and explore factors associated with level of care required.

**Methods:**

We undertook a retrospective cohort study of children <5 years old from Northern Canada transferred by urgent air transport for ARI from 2005 through 2014 to 5 pediatric tertiary care centers in Vancouver, Edmonton, Winnipeg, Ottawa and Montreal. Admissions were identified via ARI-related ICD-9/10 coding and forward sortation area. Descriptive statistics and univariable analyses were performed.

**Results:**

Among 650 urgent air transfers, the majority were from Nunavut (n = 349, 53.7%) or Nunavik (n = 166, 25.5%), <6 months old (n = 372, 57.2%), and without underlying comorbidity (n = 458; 70.5%). Estimated annual tertiary care ARI admission rates in infants <1 year old from Nunavut (40.7/1000) and Nunavik (44.5/1000) were tenfold higher than in children aged 1 to 4 years. Bronchiolitis (n = 333, 51.2%) and pneumonia (n = 208, 32.0%) were the most common primary discharge diagnoses. Nearly half required critical care (n = 316, 48.6%); mechanical ventilation rates ranged from 7.2% to 55.9% across centres. The most common primary pathogen was respiratory syncytial virus (n = 196, 30.1%). Influenza A or B was identified in 35 cases (5.4%) and vaccine-preventable bacterial infections in 27 (4.1%) cases.

**Interpretation:**

Urgent air transfers for ARI from Northern Canada are associated with high acuity. Variations in levels of care were seen across referral centers, age groups and pathogens.

## Introduction

Acute respiratory infections (ARI), namely bronchiolitis and pneumonia, are the leading causes of hospital admission and mortality in children <5 years old, worldwide [1–4]. ARI hospitalization rates in young children from circumpolar regions are significantly higher than in temperate areas of the same countries [5–7]. Infants from Inuit Nunangat, the arctic homeland of Inuit in Canada, have some of the highest ever recorded rates of ARI hospitalization, with high proportions requiring intensive care [8–10].

Most communities and villages in Inuit Nunangat are accessible only by air and are served by a local health center or nursing station. As a result, air travel for medical purposes is often inherent in accessing care, representing up to 40% of Nunavut’s healthcare budget, and is a significant contributing factor to per capita spending being more than twice the average for the rest of the country [11–14]. Regional and tertiary pediatric center admissions for ARI are expensive [15], yet cost analyses fail to capture important social and personal costs; families are separated and employment is interrupted for a caregiver to accompany their sick child. Further, it is challenging to navigate cultural and language barriers while accessing care in an unfamiliar healthcare system.

Understanding the epidemiology of ARIs in Northern Canadian populations is important because it can inform priorities for Public Health and the distribution of resources. We sought to describe patient characteristics and microbial etiologies, estimate age-specific tertiary centre admission rates, and explore factors associated with level of care for patients <5 years of age urgently transferred by air for ARI.

## Methods

### Study design

This was a multicenter, retrospective cohort study of children <5 years old from Northern Canada transferred by urgent air transport for ARI over a ten-year period (2005–2014) to centers affiliated with the Pediatric Investigators Collaborative Network on Infections in Canada. ARI was defined as an illness of <14 days’ duration with respiratory symptoms suggestive of infection. Based on the typical referral pathways of patients from Northern Canada, admissions to 5 pediatric tertiary care centers were included [16]. The typical referral pathways are from the Yukon to BC Children’s Hospital (BCCH; Vancouver, British Columbia); Kitikmeot region, Western Nunavut and the Northwest Territories to Stollery Children’s Hospital (SCH; Edmonton, Alberta); Kivalliq region, Central Nunavut to Winnipeg Children’s Hospital (WCH; Winnipeg, Manitoba); Qikiqtani region, Eastern Nunavut to Children’s Hospital of Eastern Ontario (CHEO; Ottawa, Ontario) and the three health services regions of Northern Quebec (Nunavik; Terres-Cries-de-la-Baie-James; and Nord-du-Quebec) to Montreal Children’s Hospital (MCH; Montreal, Quebec) [9].

Ethics approval was obtained from the following ethics review boards: Pediatric Panel of the Research Ethics Board of the Research Institute of the McGill University Health Centre; Children’s Hospital of Eastern Ontario Research Ethics Board; University of British Columbia Children’s and Women’s Research Ethics Board; Health Research Ethics Board, University of Manitoba; and Health Research Ethics Board, University of Alberta. The requirement of informed consent was waived by all ethics review boards. Only data relevant to this study were collected by the research teams. Data were collected, deidentified and managed using secure REDCap® electronic data capture tools hosted at McGill University Health Centre—Research Institute [17, 18]. The manuscript was reviewed by Nunavut and Nunavik community representatives who provided feedback on result interpretation and presentation.

### Case identification

Hospitalizations were identified by querying hospital discharge abstract databases using ARI-specific *International Classification of Diseases* -9 &10 codes [S1 Data] for patients admitted 2005 through 2014 (at WCH, only data from 2009–2014 were collected due to the large number of cases). Search results were narrowed by patient date of birth in relation to age at time of admission. Search results were narrowed by resident forward sortation areas (geographical unit based on the first three characters of a postal code), as related to each site’s remote Northern catchment areas. This was defined by each study site as follows: children flown in by jet from the Yukon for BCCH, the Northwest Territories or Nunavut for SCH, Nunavut for WCH, Nunavut for CHEO, and Nunavik, Terres-Cries-de-la-Baie-James and Nord-du-Quebec for MCH. Patients electively transferred, such as by scheduled flight instead of air ambulance with advanced care paramedics, or transferred for a reason other than ARI were excluded.

### Data collection

Patient paper charts were reviewed by medical students, residents, practicing physicians and research assistants. Data gathering was supervised by each site lead and included informational sessions, training, and the duplication and verification of the first 10 charts performed. Ambiguities and questions were fielded by each study site lead as they arose. In the case of indecipherable handwriting, alternate sources to corroborate data, such as referring center documentation or nursing notes were consulted. Demographics, environmental exposures, clinical history, underlying chronic comorbidities, vaccination history, microbiology of the presenting ARI, hospital course, and outcomes at discharge were collected on standardized case report forms. When more than one pathogen was identified, the site investigator adjudicated which was the likely primary pathogen based on timing, site of detection and the clinical picture. Data were de-identified, coded and entered into REDCap® by a research assistant and/or site investigator, trained by a data entry guide.

### Statistical analysis

Descriptive statistics were performed and associations between variables assessed using Chi-square test, Kruskal-Wallis test and relative risks (RR) with associated 95% confidence intervals (CI). Statistical significance was assessed using 2-tailed tests, with an α of 0.05. Annual ARI evacuation incidence rates were calculated using Statistics Canada regional population data over the years of the study for which all 5 centers provided data (2009–2014) [19–21]. Statistical analyses were performed using R version 3.5.2 (R Core Team, Vienna, Austria).

## Results

**Fig 1:**
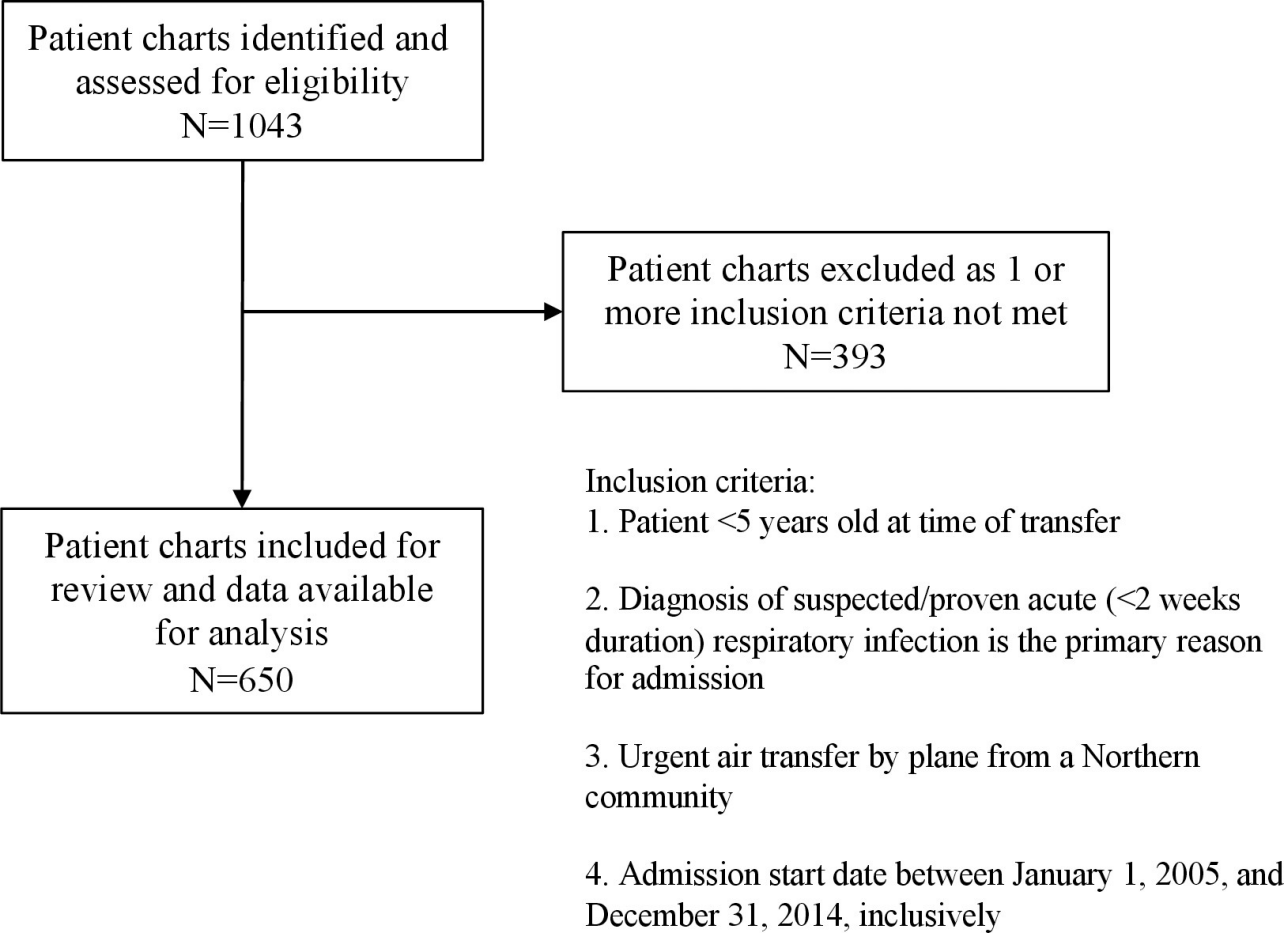
Flow diagram of patient charts.

There were 650 eligible admissions to BCCH (n = 8), SCH (n = 89), WCH (n = 194 [from 2009–2014]), CHEO (n = 93) and MCH (n = 266) [Table 1; Fig 1].

**Table 1:** Demographic characteristics of 650 children transported urgently by air from northern Canada to five tertiary care centers (n, %).

Characteristic	BCCH (N = 8)	SCH (N = 89)	WCH (N = 194)	CHEO (N = 93)	MCH (N = 266)	Total (N = 650)
Age in months						
0–5 months	6 (75.0)	43 (48.3)	104 (53.6)	57 (61.3)	162 (60.9)	372 (57.2)
6–11 months	1 (12.5)	15 (16.8)	37 (19.1)	15 (16.1)	38 (14.3)	106 (16.3)
12–23 months	1 (12.5)	21 (23.6)	39 (20.1)	14 (15.1)	48 (18.0)	123 (18.9)
24–35 months	0	1 (1.1)	6 (3.1)	4 (4.3)	7 (2.6)	18 (2.8)
36–47 months	0	5 (5.6)	5 (2.6)	3 (3.2)	10 (3.8)	23 (3.5)
48–59 months	0	4 (4.5)	3 (1.5)	0	1 (0.4)	8 (1.2)
mean (SD)	5.2 (7.0)	11.8 (14.4)	9.4 (10.6)	7.9 (9.6)	8.2 (10.2)	9.0(10.9)
median (IQR)	2.1 (1.3–4.9)	6.4 (2.5–14.4)	5.4 (2.2–12.4)	2.7 (1.8–11.3)	3.4 (1.7–11.9)	4.2(1.9–12.3)
Sex						
Male	4 (50.0)	45 (50.6)	109 (56.2)	59 (63.4)	163 (61.3)	380 (58.5)
Underlying comorbidity^1^						
None	4 (50.0)	82 (92.1)	126 (64.9)	78 (83.8)	168 (63.2)	458 (70.5)
Prematurity ≤36 weeks gestation	3 (37.5)	14 (15.7)	52 (26.8)	35 (37.6)	60 (22.6)	164 (25.2)
Significant cardiac or respiratory condition^2^	3 (37.5)	3 (3.4)	13 (6.7)	4 (4.3)	16 (6.0)	39 (6.0)
Vaccinations up to date for age						
Yes	2 (25.0)	55 (61.8)	38 (19.6)	67 (72.0)	78 (29.3)	240 (36.9)
No	2 (25.0)	34 (38.2)	156 (80.4)	26 (28.0)	188 (70.7)	406 (62.5)
Unknown	4 (50.0)	0	0	0	0	4 (0.6)
Smokers in the household						
Yes	0	15 (16.8)	112 (57.7)	67 (72.0)	78 (29.3)	272 (41.8)
No	4 (50.0)	5 (5.6)	32 (16.5)	2 (2.1)	5 (1.9)	48 (7.4)
Not recorded	4 (50.0)	69 (77.5)	50 (25.8)	24 (25.8)	183 (68.8)	330 (50.8)
Forward Sortation Area						
Nunavut (X0A, X0B, X0C)	0	54 (60.7)	194 (100)	93 (100)	8 (3.0)	349 (53.7)
RSS Nunavik (J0M)^3^	0	0	0	0	166 (62.4)	166 (25.5)
RSS Terres-Cries-de-la-Baie-James (G0W, J0Y, J0M)^4^	0	0	0	0	90 (33.8)	90 (13.8)
Northwest Territories (X0E, X0G and X1A)	0	28 (31.5)	0	0	0	28 (4.3)
Yukon (Y—)	8 (100)	4 (4.5)	0	0	0	12 (1.8)
RSS Nord du Quebec (G8P)	0	0	0	0	2 (0.8)	2 (0.3)

BCCH BC Children’s Hospital; CHEO Children’s Hospital of Eastern Ontario; IQR Interquartile range; MCH Montreal Children’s hospital; RSS Région sociosanitaires; SCH Stollery Children’s Hospital; SD Standard Deviation; WCH Winnipeg Children’s Hospital

^1^ Additional details on individual underlying comorbidities are presented in S1 Table of the Supplementary Information.

^2^ Defined as hemodynamically significant heart disease and/or chronic lung disease of prematurity.

^3^ Defined as any of the 14 communities considered as part of Nunavik (RSS17) with FSA of J0M: Akulivik, Aupaluk, Inukjuak, Ivujivik, Kangiqsualujjuaq, Kangiqsujuaq, Kangirsuk, Kuujjuaq, Kuujjuarapik, Puvirnituq, Quaqtaq, Salluit, Tasiujaq, Umiujaq.

^4^ Includes the following communities with FSAs of G0W, J0Y or J0M: Chisasibi, Eastmain, Mistissini, Nemaska, Ouje-bougoumou, Waskaganish, Waswanipi, Whapmagoostui, Wemindji that are part of the RSS 18 (Terres-Cries-de-la-Baie-James).

Overall, most patients were from Nunavut (n = 349, 53.7%) or Nunavik (n = 166, 25.5%), with the remainder from Terres-Cries-de-la-Baie-James (n = 90, 13.8%), Northwest Territories (n = 28, 4.3%) and Yukon (n = 12, 1.8%). Over half of cases (n = 372; 57.2%) were less than 6 months old, and the majority (n = 458, 70.5%) had no chronic comorbidities. Pre-existing chronic medical conditions are presented in S1 Table. There was insufficient information from chart review to reliably report household overcrowding (missingness, n = 225 [34.6%]), presence of household smokers (missingness, n = 330 [50.8%]), and family history of atopy or asthma (missingness, n = 547 [84.1%]).

Estimated annual tertiary care ARI admission rates in children <1 year old during 2009 to 2014 ranged from 2.0/1000 infants in the Northwest Territories to 44.5/1000 infants in Nunavik [Table 2]. Among patients 1–4 years old, yearly admission rates ranged from 0.1/1000 children in the Yukon to 3.9/1000 children in Nunavut. Incidence rates were not estimated for the Nord-du-Quebec region, as only 1 admission was observed during that time period.

**Table 2:** Annual tertiary care acute respiratory infection related admission rates (per 1,000 population) of children transported urgently by air from five northern Canada regions between 2009 to 2014^1^.

	Yukon (N = 9)	Northwest Territories (N = 16)	Nunavut (N = 280)	Nunavik (N = 104)	Terres-Cries-de-la-Baie-James (N = 48)
Age <1 year (95% CI)	3.3 (0.7–15.0)	2.0 (0.4–9.1)	40.7 (29.3–56.4)	44.5 (26.8–72.9)	13.5 (5.8–31.3)
Age 1–4 years (95% CI)	0.1 (0.004–2.6)	0.5 (0.1–2.4)	3.9 (2.3–6.7)	2.5 (0.8–7.3)	1.9 (0.7–5.7)

CI Confidence interval

^1^ Incidence rates were not estimated for the Nord-du-Quebec region, as only 1 admission was observed during that time period.

Admitting diagnoses and clinical manifestations are presented in S2 Table. Bronchiolitis (51.2%) and pneumonia (32.0%) were the most common primary discharge diagnoses [Table 3]. Regional Northern hospitalization, prior to transfer, varied greatly: the majority of WCH patients arrived directly from nursing stations (n = 179, 92.3%), whereas regional hospitals admitted most patients prior to transfer to SCH (n = 81, 91.0%), CHEO (n = 85, 91.4%) and MCH (n = 231, 86.8%). WCH had the lowest hospital median length of stay (4 days) and lowest proportion requiring ICU care (n = 38, 19.6%) while CHEO had the highest length of stay (13 days, p<0.001) and proportion requiring ICU care (n = 71, 76.3%, p<0.001). Overall, 48.6% of children were admitted to ICU; 31.5% received mechanical ventilation. Age <6 months (RR 1.51, 95%CI 1.04–2.21), and history of prematurity (1.25, 95%CI 1.06–1.48) were associated with ICU admission [S3 Table]. Compared with admissions with no pathogen identified, the risk of ICU admission was higher if RSV, other non-influenza respiratory viruses, *B*. *pertussis* or *H*. *influenzae* were the primary pathogen. The highest level of respiratory support varied significantly across age groups, primary pathogen identified and history of prematurity or any chronic comorbidity [S4 Table].

**Table 3:** Clinical characteristics of 650 children transported urgently by air from northern Canada to five tertiary care centers (n, %).

	BCCH (N = 8)	SCH (N = 89)	WCH (N = 194)	CHEO (N = 93)	MCH (N = 266)	Total (N = 650)
Regional hospitalization prior to transfer	1 (12.5)	81 (91.0)	15 (7.7)	85 (91.4)	231 (86.8)	420 (64.6)
Duration of symptoms prior to admission to study center (median days, IQR)	3.0 (3.0–5.5)	5.0 (2.5–11.0)	4.0 (2.0–6.0)	4.0 (3.0–6.0)	4.0 (3.0–6.0)	4.0(3.0–6.0)
Received care in ICU	3 (37.5)	58 (65.2)	38 (19.6)	71 (76.3)	146 (54.9)	316 (48.6)
Highest respiratory support						
No respiratory support	4 (50.0)	19 (21.3)	56 (28.9)	8 (8.6)	53 (19.9)	140 (21.5)
Oxygen only	2 (25.0)	31 (34.8)	108 (55.7)	27 (29.0)	85 (31.9)	253 (38.9)
Any CPAP/BiPAP	0	0	16 (8.2)	6 (6.4)	30 (11.3)	52 (8.0)
Any mechanical ventilation or HFOV	2 (25.0)	39 (43.8)	14 (7.2)	52 (55.9)	98 (36.8)	205 (31.5)
Antimicrobial therapy	NA^1^	80 (89.9)	142 (73.2)	86 (92.5)	218 (82.0)	526 (80.9)
Procedures done at study site						
Lumbar puncture	0	9 (10.1)	24 (12.4)	32 (34.4)	37 (13.9)	102 (15.7)
Procedure requiring general anesthesia	0	5 (5.6)	0	10 (10.8)	40 (15.0)	55 (8.5)
Tube thoracostomy	0	8 (9.0)	0	5 (5.4)	14 (5.3)	27 (4.1)
Thoracentesis	0	1 (1.1)	0	1 (1.1)	9 (3.4)	11 (1.7)
Length of stay at tertiary care center (days)						
Mean (SD)	7.9 (7.4)	13.4 (13.9)	5.4 (6.2)	18.4 (18.5)	14.2 (19)	12.0 (16.0)
Median (IQR)	5.0 (2.0–12.0)	10.0 (5.0–15.0)	4.0 (2.0–7.0)	13.0 (9.0–22.0)	9.0 (5.0–18.0)	7.0 (4.0–14.0)
Primary discharge diagnosis						
Bronchiolitis	6 (75.0)	41 (46.1)	102 (52.6)	56 (60.2)	128 (48.1)	333 (51.2)
Pneumonia	0	24 (27.0)	88 (45.4)	19 (20.4)	77 (28.9)	208 (32.0)
Other	2 (25.0)	19 (21.3)	2 (1.0)	10 (10.7)	52 (19.5)	85 (13.1)
Apnea	0	2 (2.2)	0	5 (5.4)	5 (1.9)	12 (1.8)
Asthma/reactive airway disease	0	1 (1.1)	1 (0.5)	2 (2.2)	3 (1.1)	7 (1.1)
Empyema	0	1 (1.1)	0	1 (1.1)	0	2 (0.3)
Sepsis/shock	0	0	1 (0.5)	0	1 (0.4)	2 (0.3)
Pleural effusion	0	1 (1.1)	0	0	0	1 (0.1)
Previous admissions to study centre						
Yes	3 (37.5)	14 (15.7)	49 (25.3)	10 (10.8)	53 (19.9)	129 (19.8)
Not recorded	0	29 (32.6)	2 (1.0)	0	2 (0.8)	33 (5.1)
Final disposition						
Discharged home	6 (75.0)	61 (68.5)	194 (100.0)	93 (100.0)	262 (98.5)	616 (94.8)
Transfer to other hospital	2 (25.0)	19 (21.3)	0	0	1 (0.4)	22 (3.4)
Transfer to long-term care facility/rehab	0	7 (7.9)	0	0	0	7 (1.1)
Death in hospital	0	1 (1.1)	0	0	2 (0.8)	3 (0.5)
Unknown	0	1 (1.1)	0	0	0	1 (0.1)

BCCH BC Children’s Hospital; BiPAP Bilevel Positive Airway Pressure; CHEO Children’s Hospital of Eastern Ontario; CPAP Continuous Positive Airway Pressure; CPR Cardiopulmonary resuscitation; ECMO Extracorporeal membrane oxygenation; HFOV High Frequency Oscillatory Ventilation; ICU Intensive care unit; IQR Interquartile range; MCH Montreal Children’s hospital; NA Not available; SCH Stollery Children’s Hospital; SD Standard Deviation; WCH Winnipeg Children’s Hospital

^1^ Data on antimicrobial use was not collected at BCCH.

**Fig 2:**
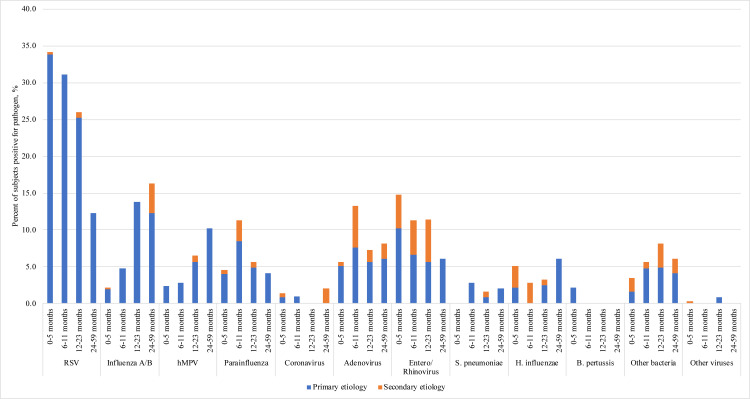
Identified pathogens by age group, and primary or secondary infectious etiology. Other bacteria: For primary etiology: *M*. *catarrhalis* n = 1/1/0/0; *Acetinobacter spp* n = 0/1/1/0; *C*. *trachomatis* n = 1/0/0/0; *S*. *viridans* n = 1/0/1/0; MRSA n = 1/0/1/1; MSSA n = 0/0/1/0; Group A strep n = 1/3/1/1; Group B strep n = 1/0/1/0; For secondary etiology: *M*. *catarrhalis* n = 6/0/3/1; MRSA n = 1/0/1/0; TB n = 0/1/0/0. Other viruses: For primary etiology: Cytomegalovirus n = 0/0/1/0; For secondary etiology: bocavirus n = 1/0/0/0.

At least one potential etiologic agent was detected in 430 cases (66.1%) [Table 4]. Fig 2 presents the distribution of identified primary and secondary pathogens across age groups. Overall, RSV was the most common primary pathogen (n = 196, 30.1%) and was detected approximately three times more frequently in children <6 months of age (n = 126, 33.9%) than in those 2–4 years old (n = 6, 12.2%). Influenza A or B were detected in 5.4% (n = 35) of cases. Other non-influenza respiratory viruses were the primary pathogen in 23.5% (n = 153). Potentially vaccine-preventable bacteria (*S*. *pneumoniae*, *H*. *influenzae* and *B*. *pertussis*) were the primary pathogens in 27 cases (4.1%). Overall, there was more than one laboratory confirmed infection identified in 13.1% (n = 85) of admissions. Although laboratory-confirmed bacterial infection was uncommon (46 primary infections [7.1%]; 29 secondary infections [4.5%]), 8 out of every 10 cases were treated with antibiotics.

**Table 4:** Microbiology results of 650 children transported urgently by air from northern Canada to five tertiary care centers (n, %).

	BCCH (N = 8)	SCH (N = 89)	WCH (N = 194)	CHEO (N = 93)	MCH (N = 266)	Total (N = 650)
Lab confirmation of etiologic agent	6 (75.0)	71 (79.8)	104 (53.6)	50 (53.8)	199 (74.8)	430 (66.1)
Primary pathogen identified						
RSV	3 (37.5)	28 (31.4)	50 (25.8)	28 (30.1)	87 (32.7)	196 (30.1)
Adenovirus	0	7 (7.9)	13 (6.7)	2 (2.2)	15 (5.6)	37 (5.7)
Rhinovirus	0	0	8 (4.1)	0	26 (9.8)	34 (5.2)
Parainfluenza virus	0	6 (6.7)	8 (4.1)	2 (2.2)	16 (6.0)	32 (4.9)
Type 1	0	0	2 (1.0)	0	4 (1.5)	6 (0.9)
Type 2	0	0	0	0	0	0
Type 3	0	1 (1.1)	5 (2.6)	2 (2.2)	11 (4.1)	19 (2.9)
Type 4	0	0	0	0	0	0
Influenza A	0	6 (6.7)	6 (3.1)	4 (4.3)	9 (3.4)	25 (3.8)
H1N1 subtype	0	5 (5.6)	4 (2.1)	1 (1.1)	4 (1.5)	14 (2.1)
H3N2 subtype	0	0	0	0	2 (0.8)	2 (0.3)
hMPV	1 (12.5)	2 (2.2)	5 (2.6)	5 (5.4)	11 (4.1)	24 (3.7)
*H*. *influenzae*	1 (12.5)	3 (3.4)	1 (0.5)	3 (3.2)	6 (2.2)	14 (2.1)
Rhinovirus/Enterovirus	0	11 (12.4)	1 (0.5)	1 (1.1)	0	13 (2.0)
Influenza B	0	0	4 (2.1)	0	6 (2.3)	10 (1.5)
Enterovirus	0	0	1 (0.5)	2 (2.2)	5 (1.9)	8 (1.2)
*B*. *pertussis*	0	1 (1.1)	1 (0.5)	2 (2.2)	4 (1.5)	8 (1.2)
Other bacteria	1 (12.5)^1^	2 (2.2)^2^	1 (0.5)^3^	0	3 (1.1)^4^	7 (1.1)
Group A strep	0	2 (2.2)	0	1 (1.1)	3 (1.1)	6 (0.9)
*S*. *pneumoniae*	0	0	2 (1.0)	0	3 (1.1)	5 (0.8)
Coronavirus	0	1 (1.1)	2 (1.0)	0	1 (0.4)	4 (0.6)
MRSA	0	1 (1.1)	1 (0.5)	0	1 (0.4)	3 (0.5)
Group B strep	0	0	0	0	2 (0.8)	2 (0.3)
MSSA	0	1 (1.1)	0	0	0	1 (0.1)
Other organism	0	0	0	0	1 (0.4)^5^	1 (0.1)
TB	0	0	0	0	0	0
Laboratory confirmed concurrent infection	0	17 (19.1)	23 (11.8)	8 (8.6)	37 (13.9)	85 (13.1)

B Bordetella; BCCH BC Children’s Hospital; CHEO Children’s Hospital of Eastern Ontario; H Haemophilus; hMPV human metapneumovirus; IQR Interquartile range; MCH Montreal Children’s hospital; MSSA methicillin-sensitive staphylococcus aureus; MRSA methicillin-resistant Staphylococcus aureus; RSV Respiratory Syncytial Virus; S Streptococcus; SCH Stollery Children’s Hospital; SD Standard Deviation; strep streptococcus; TB tuberculosis; WCH Winnipeg Children’s Hospital

^1^ *Acetinobacter spp* (n = 1)

^2^ *M*. *catarrhalis* (n = 2)

^3^ *Acetinobacter spp*.(n = 1)

^4^ *S*. *viridans* (n = 2), *C*. *trachomatis* (n = 1)

^5^ Cytomegalovirus (n = 1)

## Discussion

This is the first Canada-wide collaborative epidemiologic study of pediatric tertiary care center ARI admissions from Northern Canada. We found very high yearly admission rates in both Nunavut (40.7 per 1000 infants) and Nunavik (44.5 per 1000 infants) among children less than one year of age, which were tenfold higher than for children 2–4 years old. Tertiary care admission rates from the Yukon and Northwest Territories were comparatively much lower, at 3.3 per 1000 infants and 2.0 per 1000 infants yearly, respectively [22]. Our estimated hospitalization rates represent the subset of patients with the highest ARI severity that require urgent air transfer to a pediatric tertiary care center; as such, they are predictably smaller than previously reported staggeringly high *regional* ARI admission rates in Nunavut of 306 to 590 per 1000 infants less than one year of age [5, 8, 9, 23]. In comparison, North American bronchiolitis admission rates overall have trended down to 11.8–13.5 per 1000 infants less than one year of age [24, 25]. Similarly, Australian aboriginal children and Alaska Native infants also experience a several factor-fold higher rate of admission for ARIs compared to national rates for the same age groups [7, 26].

Admission rates in this study reflect a spectrum of disease severity and merit descriptive nuance. First, we observed significantly different admission profiles between the five tertiary care centers involved in our study. Whereas the vast majority of WCH admissions were transferred directly from nursing stations to WCH without intermediate regional hospitalization, the MCH receives transfers from regional hospitals staffed mainly by primary care providers with consulting paediatricians. The significantly higher number of admissions to WCH and less severe case-mix are likely a reflection of infrequent regional hospitalization prior to transfer. The lack of regional care is an increasing trend in North America and leads to transfer as a rule, with loss of regional pediatric capacity and little room to surge [27]. SCH and CHEO’s transfers generally hail from larger centers, Stanton Territorial Hospital (STH) in Yellowknife and Qikiqtani General Hospital (QGH) in Iqaluit, that have benefitted from improved access to on-site care from full-time pediatricians. This specialized care in a general hospital likely decreases the overall number of transfers, but results in an increased proportion of transfers that require PICU admission and invasive mechanical ventilation. A qualitative study in Nunavut has suggested that transfers reflect both the referring and receiving providers’ experience and the staffing in health centres, with there being challenges related to recruitment and retention of staff [28]. The availability of specialized care, including diagnostic tests and treatments such as early provision of non-invasive respiratory support, could reduce the need for transfer to a Southern referral center [29]. With the expanding role of telemedicine, which has benefitted from much greater uptake and technological developments since the start of the COVID-19 pandemic, this could alleviate some stress on staffing issues. On the other hand, the very few admissions from the Yukon may reflect intermediate hospitalization at Whitehorse General Hospital, which has paediatrician coverage, as well as a more urban and lower risk population. Nevertheless, when taken as a whole, these transfers from Northern Canada represent severe cases of ARI, with nearly half (48.6%) of the children in the cohort requiring admission to ICU and very high rates of invasive mechanical ventilation (31.5%). In other parts of North America, Europe and Australia, rates of intubation and mechanical ventilation for young children admitted with bronchiolitis or pneumonia are consistently far lower at 1.6%-7% [3, 25, 30–33].

The reason for the exceptional ARI burden in this population is likely multifactorial. There are demonstrated relationships between LRTIs and social determinants of health, including high rates of overcrowding in homes, poor indoor air quality, remote residence, and smoking during pregnancy [6, 10, 34–37]. Due to the retrospective nature of this study, it was not possible to collect accurate data on ethnicity and on social and environmental risk factors. Additionally, many children arrived at tertiary care centers unaccompanied by parents due to historical policies that prevented parents from boarding medical evacuation flights [38] which further impaired collection of such information. Data available through the 2018 Inuit Statistical profile indicates that 52% of Inuit homes are overcrowded, 70% households are food insecure and 63% of residents 12 years of age and older report daily cigarette smoking [39]. Given the enormous impact of the social determinants of health, this is an aspect of medical history taking and care that requires emphasis.

A primary pathogen was microbiologically identified in approximately two-thirds of patients with about one-third having RSV. Among infants less than 6 months of age, 33.9% (n = 126) had RSV, as did 31.1% (n = 33) of those between 6 and 11 months. This proportion is comparable to published data from infants admitted for bronchiolitis in Northern Canada [5, 23, 40] or for community-acquired pneumonia in the United States [3] and in low-middle income countries [2]. Although there is no licensed vaccine for RSV, passive immunoprophylaxis with palivizumab has been shown to be safe and effective at preventing RSV-associated hospitalization in specific high-risk infant groups [41, 42]. Drawbacks of an immunoprophylaxis strategy include the high cost ($5,000-$9,000 per infant per year), monthly injections during RSV season [43], in addition to the administrative challenge of identifying and tracking eligible patients [44]. However, it has been estimated that direct costs of admission to Qikiqtani General Hospital in Nunavut averaged $14,273 CAD in 2005, and a transfer to CHEO in Ottawa costs upwards of $45,000 CAD [45–47]. Two Canadian cost-effectiveness analyses have suggested that universal palivizumab prophylaxis for infants <6 months of age would be cost-saving in some, but not all, rural Arctic communities [44] and the Canadian Pediatric Society stated that palivizumab immunoprophylaxis may be considered in term Inuit infants <6 months of age during RSV season if they live in communities with documented persistent high rates of RSV hospitalization. Unfortunately, theory has not translated well to practice: in Quebec, from 2016–2017 through to 2019–2020, the provincial program was expanded to include healthy term Nunavik infants <3 months old and in the three seasons studied, there was no evidence that palivizumab reduced RSV hospitalizations in that population [48]. Preventing RSV infection in young children living in remote settings has been identified as a public health priority by the Public Health Agency of Canada [49]. As roughly two-thirds of urgent air transfers for ARI in this population appear to be due to pathogens other than RSV, strategies to address ARI prevention more broadly are sorely needed, such as optimizing routine vaccination coverage, smoking cessation, reducing household crowding, improving food security and timely access to appropriate diagnosis and management. Indeed, the COVID-19 pandemic has demonstrated that non-pharmacological interventions to slow SARS-COV-2 transmission also significantly reduced the overall rates of emergency department visits and hospitalizations for pediatric infectious illnesses [50–53].

Regarding other potentially vaccine-preventable illnesses, pertussis was uncommon (8 cases identified) but associated with need for ICU admission (100%) and mechanical ventilation (50%). Influenza was infrequently detected in infants (<5%), but was associated with 13.8% (n = 17) and 12.2% (n = 6) of admissions in the 12–23 month-old and 24–59 month-old age brackets, respectively. The report of an up-to-date vaccination schedule varied widely in subjects from different study sites (19.6% to 72.0%). This highlights an area of inexpensive, preventable illness that can get lost with barriers to care, such as a pandemic, geographical or weather-induced isolation and the need for research into optimizing vaccination scheduling and adherence in this vulnerable population [54, 55].

This study has several limitations. Patients deceased prior to transfer would have been missed, as would patients that deviated from habitual transfer routes due to weather or bed-space limitations. Further, the retrospective nature of the data collection makes gathering information about the social history difficult. Previous palivizumab receipt may not have been recorded in hospital charts, especially for infants in a non-traditional high risk group. Microbiologic testing was not uniformly performed; in particular, the availability of highly-multiplexed PCR panels, the gold standard for respiratory virus diagnostics [56, 57], varied over time and by site.

In conclusion, urgent air transfers for severe ARI in infants from Northern Canada are common and are associated with a high level of acuity, including need for critical care and mechanical ventilation, especially in children <1 year-old. This disease burden is likely a reflection of access to local care and resources, but also of social inequity and socio-economic status. Urgent attention to this topic is warranted and there is a pressing need for public health strategies and interventions to decrease the risk of severe ARI and improve the health of young children in remote settings.

## Supporting information

S1 Table Underlying chronic medical conditions (n, %). (DOCX)

S2 Table Admitting diagnoses and presenting manifestations (n, %). (DOCX)

S3 Table Intensive care unit requirements by age group, primary pathogen, and comorbidity. (DOCX)

S4 Table Highest respiratory support received by age group, primary pathogen, and comorbidity. (DOCX)

S5 Table Urgent air transfers to four tertiary care centers by region in Nunavut (n, %). (DOCX)

S1 Data ICD-10 Diagnostic codes related to ARI. (DOCX)
