# Supplementary material for: Urgent air transfers for acute respiratory infections among children from Northern Canada, 2005–2014
Source: PLoS One. 2022 Jul 28;17(7):e0272154. doi: 10.1371/journal.pone.0272154 (PMC9333212; doi:10.1371/journal.pone.0272154)
Supplement: S1 Table — (DOCX) [file pone.0272154.s001.docx]

# S1 Table. Underlying chronic medical conditions (n, %)

|  | **BCCH**  (N=8) | **SCH**  (N=89) | **WCH**  (N=194) | **CHEO**  (N=93) | **MCH**  (N=266) | **Total**  (N=650) |
| --- | --- | --- | --- | --- | --- | --- |
| No chronic condition | 4  (50.0) | 82  (92.1) | 126 (64.9) | 78  (83.9) | 168 (63.2) | 458  (70.5) |
| Any chronic condition | 4  (50.0) | 7  (7.9) | 68  (35.1) | 15  (16.1) | 98  (36.8) | 192  (29.5) |
| Prematurity <36 weeks gestation | 3 (37.5) | 14 (15.7) | 52 (26.8) | 35 (37.6) | 60 (22.6) | 164 (25.2) |
| Other chronic lung disease^1^ | 1 (12.5) | 1 (1.1) | 11 (5.7) | 3 (3.2) | 30 (11.3) | 46 (7.1) |
| Other cardiac findings^2^ | 0 | 2 (2.2) | 12 (6.2) | 5 (5.3) | 21 (7.8) | 40 (6.1) |
| Other multi-system disorder/syndrome^3^ | 0 | 0 | 25 (12.9) | 0 | 8 (3.0) | 33 (5.1) |
| Neurological/developmental disorder with impaired handling of secretions | 0 | 0 | 4 (2.1) | 0 | 21 (7.9) | 25 (3.8) |
| Congenital heart disease or lesion of hemodynamic significance | 2 (25.0) | 2 (2.2) | 8 (4.1) | 2 (2.1) | 10 (3.8) | 24 (3.7) |
| Hematologic disorder, non-malignant^4^ | 0 | 0 | 0 | 1 (1.1) | 21 (7.9) | 22 (3.4) |
| Other chronic condition | 1 (12.5)^5^ | 3 (3.3)^6^ | 5 (2.6)^7^ | 3 (3.2)^8^ | 8 (3.0)^9^ | 20 (3.1) |
| Other neurological disorders^10^ | 0 | 0 | 4 (2.1) | 4 (4.3) | 10 (3.8) | 18 (2.8) |
| Lung disease of prematurity | 1 (12.5) | 1 (1.1) | 6 (3.1) | 3 (3.2) | 6 (2.3) | 17 (2.6) |
| Gastrointestinal/hepatic disorder^11^ | 0 | 0 | 4 (2.1) | 0 | 12 (4.5) | 16 (2.5) |
| Airway anomaly^12^ | 0 | 1 (1.1) | 1 (0.5) | 0 | 9 (3.4) | 11 (1.7) |
| Genitourinary/renal disorder^13^ | 0 | 0 | 1 (0.5) | 0 | 2 (0.7) | 3 (0.5) |
| Endocrine/nutritional/metabolic disorder | 0 | 0 | 0 | 0 | 2 (0.7)^14^ | 2 (0.3) |
| Immunosuppression | 1 (12.5)^15^ | 0 | 0 | 0 | 0 | 1 (0.1) |

BCCH BC Children’s Hospital; CHEO Children’s Hospital of Eastern Ontario; MCH Montreal Children’s hospital; SCH Stollery Children’s Hospital; WCH Winnipeg Children’s Hospital

^1^ asthma, reactive airway disease, chronic cough.

^2^ atrial septal defect, ventricular septal defect, aortic insufficiency, tricuspid regurgitation.

^3^ CPT1 deficiency, Neurofibromatosis type 1, Goldenhar Syndrome, lysosomal storage disease, Greig’s syndrome, HyperIgE syndrome, Tetrasomy 4, Fetal Alcohol Syndrome, microdeletion Chromosome 7.

^4^ anemia, thrombocytopenia.

^5^ failure to thrive.

^6^ failure to thrive, chromosomal duplication, Wolf-Parkinson-White.

^7^ morbid obesity, optic glioma, failure to thrive.

^8^ multicystic dysplastic kidneys, severe atopic dermatitis and anaphylactic allergies, cutis laxa.

^9^ situs inversus totalis, ventriculo-peritoneal shunt, failure to thrive.

^10^ hypoxic ischemic encephalopathy, periventricular leukomalacia, controlled epilepsy, hypotonia, cerebral palsy, white matter disease, infantile seizure disorder NYD, agenesis of corpus callosum.

^11^ repaired biliary atresia, repaired gastroschisis, giant cell hepatitis, hyperbilirubinemia NYD, multiple bowel obstructions repaired, hepatitis, hepatitis with cholestasis NYD, metabolic disorder not otherwise specified.

^12^ vocal cord paralysis, tracheomalacia, bronchomalacia, laryngeal cleft, subglottic stenosis.

^13^ renal calcifications, hydronephrosis.

^14^ rickets.

^15^ DiGeorge syndrome.
