# Supplementary material for: Urgent air transfers for acute respiratory infections among children from Northern Canada, 2005–2014
Source: PLoS One. 2022 Jul 28;17(7):e0272154. doi: 10.1371/journal.pone.0272154 (PMC9333212; doi:10.1371/journal.pone.0272154)
Supplement: S2 Table — (DOCX) [file pone.0272154.s002.docx]

# S2 Table. Admitting diagnoses and presenting manifestations (n, %)

|  | **BCCH**  (N=8) | **SCH**  (N=89) | **WCH**  (N=194) | **CHEO**  (N=93) | **MCH**  (N=266) | **Total**  (N=650) |
| --- | --- | --- | --- | --- | --- | --- |
| Primary admitting diagnosis |  |  |  |  |  |  |
| Bronchiolitis | 7 (87.5) | 28 (31.5) | 94 (48.5) | 33 (35.5) | 73 (27.4) | 235  (36.1) |
| Pneumonia | 0 | 19 (21.3) | 82 (42.3) | 14 (15.1) | 56 (21.1) | 171  (26.3) |
| Respiratory distress/difficulty breathing | 1 (12.5) | 17 (19.1) | 7 (3.6) | 22 (23.7) | 70 (26.3) | 117  (18.0) |
| Apnea | 0 | 3 (3.4) | 1 (0.5) | 19 (20.4) | 13 (4.9) | 36 (5.5) |
| Other | 0 | 14 (15.7) | 1 (0.5) | 0 | 21 (7.9) | 36 (5.5) |
| Respiratory failure | 0 | 4 (4.5) | 4 (2.1) | 2 (2.2) | 19 (7.1) | 29 (4.5) |
| Asthma/ wheezing/  reactive airway disease | 0 | 2 (2.2) | 2 (1.0) | 2 (2.2) | 3 (1.1) | 9 (1.4) |
| Sepsis / shock | 0 | 0 | 2 (1.0) | 0 | 6 (2.3) | 8 (1.2) |
| ALTE | 0 | 0 | 1 (0.5) | 1 (1.1) | 4 (1.5) | 6 (0.9) |
| Pleural effusion | 0 | 1 (1.1) | 0 | 0 | 0 | 1 (0.1) |
| Empyema | 0 | 1 (1.1) | 0 | 0 | 0 | 1 (0.1) |
| Fever without source | 0 | 0 | 0 | 0 | 1 (0.4) | 1 (0.1) |
| Other diagnoses contributing to transfer |  |  |  |  |  |  |
| Respiratory distress/difficulty breathing | 0 | 3 (3.4) | 15 (7.7) | 33 (35.5) | 66 (24.8) | 117  (18.0) |
| Pneumonia | 0 | 7 (7.9) | 29 (14.9) | 19 (20.4) | 49 (18.4) | 104  (16.0) |
| Bronchiolitis | 0 | 5 (5.6) | 20 (10.3) | 14 (15.1) | 39 (14.7) | 78 (12.0) |
| Sepsis/Shock | 0 | 0 | 12 (6.2) | 19 (20.4) | 6 (2.3) | 37 (5.7) |
| Respiratory failure | 0 | 0 | 6 (3.1) | 13 (14.0) | 15 (5.6) | 34 (5.2) |
| Apnea | 0 | 1 (1.1) | 4 (2.1) | 12 (12.9) | 11 (4.1) | 28 (4.3) |
| Asthma/wheezing/reactive airway disease | 0 | 0 | 5 (2.6) | 5 (5.4) | 16 (6.0) | 26 (4.0) |
| Pleural effusion | 0 | 2 (2.2) | 1 (0.5) | 6 (6.5) | 9 (3.4) | 18 (2.8) |
| Empyema | 0 | 5 (5.6) | 0 | 0 | 2 (0.8) | 7 (1.1) |
| Fever without source | 0 | 0 | 0 | 0 | 6 (2.3) | 6 (0.9) |
| ALTE | 0 | 0 | 1 (0.5) | 1 (1.1) | 1 (0.4) | 3 (0.5) |
| Presenting manifestations of current illness |  |  |  |  |  |  |
| Cough | 7 (87.5) | 59 (66.3) | 174 (89.7) | 75 (80.6) | 188 (70.7) | 503  (77.4) |
| Increased work of breathing | 7 (87.5) | 55 (61.8) | 162 (83.5) | 73 (78.5) | 153 (57.5) | 450  (69.2) |
| Fever | 2 (25.0) | 39 (43.8) | 135 (69.6) | 53 (57.0) | 151 (56.8) | 380  (58.5) |
| Rhinorrhea/upper airway secretions | 5 (62.5) | 32 (36.0) | 96 (49.5) | 63 (67.7) | 149 (56.0) | 345  (53.1) |
| Poor feeding | 2 (25.0) | 12 (13.5) | 71 (36.6) | 39 (41.9) | 88 (33.1) | 212  (32.6) |
| Wheezing | 0 | 11 (12.4) | 65 (33.5) | 11 (11.8) | 48 (18.0) | 135  (20.8) |
| Vomiting | 1 (12.5) | 12 (13.5) | 41 (21.1) | 17 (18.3) | 49 (18.4) | 120  (18.5) |
| Apnea | 1 (12.5) | 11 (12.4) | 24 (12.4) | 38 (40.9) | 40 (15.0) | 114  (17.5) |
| Lethargy | 0 | 12 (13.5) | 32 (16.5) | 20 (21.5) | 33 (12.4) | 97 (14.9) |
| Diarrhea | 0 | 10 (11.2) | 32 (16.5) | 4 (4.3) | 24 (9.0) | 70 (10.8) |
| Dehydration | 1 (12.5) | 0 | 22 (11.3) | 8 (8.6) | 14 (5.3) | 45 (6.9) |
| Seizures | 0 | 3 (3.4) | 3 (1.5) | 4 (4.3) | 10 (3.8) | 20 (3.1) |
| Otalgia | 0 | 0 | 3 (1.5) | 3 (3.2) | 12 (4.5) | 18 (2.8) |
| Sore throat | 0 | 0 | 3 (1.5) | 2 (2.2) | 5 (1.9) | 10 (1.5) |
| Altered level of consciousness | 1 (12.5) | 1 (1.1) | 0 | 1(1.1) | 6 (2.3) | 9 (1.4) |
| ALTE | 0 | 0 | 3 (1.5) | 6 (6.5) | 0 | 9 (1.4) |
| Cardiac arrest | 0 | 0 | 0 | 2 (2.2) | 1 (0.4) | 3 (0.5) |
| Septic shock | 0 | 0 | 1 (0.5) | 0 | 0 | 1 (0.1) |
| Acute kidney injury | 0 | 0 | 0 | 0 | 1 (0.4) | 1 (0.1) |
| Admission unit |  |  |  |  |  |  |
| Medical ward | 6 (75.0) | 31 (34.8) | 169 (87.1) | 23 (24.7) | 134 (50.4) | 363  (55.8) |
| PICU | 2 (25.0) | 54 (60.7) | 25 (12.9) | 69 (74.2) | 127 (47.7) | 277  (42.6) |
| NICU | 0 | 2 (2.2) | 0 | 1 (1.1) | 5 (1.9) | 8 (1.2) |
| Missing | 0 | 2 (2.2) | 0 | 0 | 0 | 2 (0.3) |

ALTE Apparent life-threatening event; BCCH BC Children’s Hospital; CHEO Children’s Hospital of Eastern Ontario; MCH Montreal Children’s hospital; NICU neonatal intensive care unit; PICU pediatric intensive care unit; SCH Stollery Children’s Hospital; WCH Winnipeg Children’s Hospital
