# Supplementary material for: Urgent air transfers for acute respiratory infections among children from Northern Canada, 2005–2014
Source: PLoS One. 2022 Jul 28;17(7):e0272154. doi: 10.1371/journal.pone.0272154 (PMC9333212; doi:10.1371/journal.pone.0272154)
Supplement: S3 Table — (DOCX) [file pone.0272154.s003.docx]

# S3 Table. Intensive care unit requirements by age group, primary pathogen, and comorbidity

|  | **No ICU**  **N=334 (%)^1^** | **ICU**  **N=316 (%)^1^** | **Total**  **N=650** | **RR**  **(95% CI)** | **P value^2^** |
| --- | --- | --- | --- | --- | --- |
| Type of care unit by age group |  |  |  |  |  |
| 0-5 | 165 (44.4) | 207 (55.6) | 372 | 1.51  (1.04-2.21) | 0.015 |
| 6-11 | 61 (57.5) | 45 (42.4) | 106 | 1.15  (0.75-1.77) | 0.598 |
| 12-23 | 77 (62.6) | 46 (37.4) | 123 | 1.02  (0.66-1.57) | 1.00 |
| 24-59 | 31 (63.3) | 18 (36.7) | 49 | Ref. | Ref. |
| Type of care unit by primary pathogen |  |  |  |  |  |
| No organism identified | 143 (65.0) | 77 (35.0) | 220 | Ref. | Ref. |
| RSV | 86 (43.9) | 110 (56.1) | 196 | 1.60  (1.29-1.99) | <0.001 |
| Other viruses | 70 (45.7) | 83 (54.2) | 153 | 1.55  (1.23-1.95) | <0.001 |
| Influenza A/B | 22 (62.9) | 13 (37.1) | 35 | 1.06  (0.66-1.69) | 0.850 |
| Other bacteria | 8 (42.1) | 11 (57.9) | 19 | 1.65  (1.08-2.53) | 0.080 |
| *H. influenzae* | 2 (14.3) | 12 (85.7) | 14 | 2.45  (1.85-3.24) | <0.001 |
| *B. pertussis* | 0 | 8 (100.0) | 8 | 2.86  (2.39-3.42) | <0.001 |
| *S. pneumoniae* | 3 (60.0) | 2 (40.0) | 5 | 1.14  (0.38-3.39) | 1.00 |
| Prematurity <36 weeks gestation | 70 (42.7) | 94 (57.3) | 164 | 1.25  (1.06-1.48) | 0.011 |
| Significant cardiac or respiratory condition^3^ | 18 (46.1) | 21 (53.8) | 39 | 1.11  (0.82-1.51) | 0.514 |

CI confidence interval; ICU intensive care unit; Ref., reference category; RR risk ratio; RSV respiratory syncytial virus

^1^ Percentages were calculated for the row, i.e., % of a stratum requiring ICU care or not.

^2^ P values represent the following: each stratum is compared to the exposure reference level and test of independence two-sided p values are calculated using Fisher exact or chi-squared test, as appropriate.

^3^ Defined as hemodynamically significant heart disease and/or chronic lung disease of prematurity.
