# Supplementary material for: Urgent air transfers for acute respiratory infections among children from Northern Canada, 2005–2014
Source: PLoS One. 2022 Jul 28;17(7):e0272154. doi: 10.1371/journal.pone.0272154 (PMC9333212; doi:10.1371/journal.pone.0272154)
Supplement: S4 Table — (DOCX) [file pone.0272154.s004.docx]

# S4 Table. Highest respiratory support received by age group, primary pathogen, and comorbidity

|  | **No respiratory support**  **N=140 (%)^1^** | **Oxygen only**  **N=253 (%)^1^** | **CPAP/**  **BiPAP alone or with oxygen**  **N=52 (%)^1^** | **Any mechanical ventilation, HFOV**  **N=205 (%)^1^** | **Total**  **N=650** | **P value** |
| --- | --- | --- | --- | --- | --- | --- |
| Age group |  |  |  |  |  | <0.001 |
| 0-5 | 60 (16.1) | 138 (37.1) | 34 (9.1) | 140 (37.6) | 372 |  |
| 6-11 | 24 (22.6) | 48 (45.3) | 7 (6.6) | 27 (25.5) | 106 |  |
| 12-23 | 38 (30.9) | 49 (39.8) | 9 (7.3) | 27 (21.9) | 123 |  |
| 24-59 | 18 (36.7) | 18 (36.7) | 2 (4.1) | 11 (22.4) | 49 |  |
| Primary pathogen |  |  |  |  |  |  |
| No organism identified | 70 (31.8) | 92 (41.8) | 15 (6.8) | 43 (19.5) | 220 | <0.001 |
| Other viruses^2^ | 33 (21.6) | 50 (32.7) | 16 (10.5) | 54 (35.3) | 153 |  |
| RSV | 21 (10.7) | 82 (41.8) | 19 (9.7) | 74 (37.8) | 196 |  |
| Influenza A/B | 9 (25.7) | 16 (45.8) | 1 (2.9) | 9 (25.7) | 35 |  |
| Other bacteria^3^ | 1 (5.3) | 8 (42.1) | 0 | 10 (52.6) | 19 |  |
| *H. influenzae* | 3 (21.4) | 1 (7.1) | 0 | 10 (71.4) | 14 |  |
| *B. pertussis* | 1 (12.5) | 2 (25.0) | 1 (12.5) | 4 (50.0) | 8 |  |
| *S. pneumoniae* | 2 (40.0) | 2 (40.0) | 0 | 1 (20.0) | 5 |  |
| Presence of any underlying comorbidity | 49 (25.5) | 79 (41.1) | 20 (10.4) | 44 (22.9) | 192 | 0.013 |
| History of prematurity <36 weeks gestation | 24 (14.6) | 62 (37.8) | 14 (8.5) | 64 (39.0) | 164 | 0.026 |
| Significant cardiac or respiratory condition^4^ | 5 (12.8) | 18 (46.1) | 4 (10.3) | 12 (30.8) | 39 | 0.515 |

CPAP Continuous Positive Airway Pressure; CPR Cardiopulmonary resuscitation; ECMO Extracorporeal membrane oxygenation; HFOV High Frequency Oscillatory Ventilation; RSV respiratory syncytial virus

^1^ Percentages were calculated for the row, i.e., % of a stratum receiving each level of respiratory support.

^2^ Adenovirus, coronavirus, cytomegalovirus, human metapneumovirus, parainfluenza, rhinovirus, enterovirus.

^3^ Acinetobacter spp, *C. trachomatis*, group A streptococcus, group B streptococcus, *M. catarrhalis*, *S. aureus*, MRSA, *S. viridans*, *M. tuberculosis.*

^4^ Defined as hemodynamically significant heart disease and/or chronic lung disease of prematurity.
