# Supplementary material for: Urgent air transfers for acute respiratory infections among children from Northern Canada, 2005–2014
Source: PLoS One. 2022 Jul 28;17(7):e0272154. doi: 10.1371/journal.pone.0272154 (PMC9333212; doi:10.1371/journal.pone.0272154)
Supplement: S5 Table — (DOCX) [file pone.0272154.s005.docx]

# S5 Table. Urgent air transfers to four tertiary care centers by region in Nunavut (n, %)

|  | **SCH**  (N=54) | **WCH**  (N=194) | **CHEO**  (N=93) | **MCH**  (N=8) | **Total**  (N=349) |
| --- | --- | --- | --- | --- | --- |
| Region of Nunavut |  |  |  |  |  |
| Qikiqtaaluk | 1 (1.8) | 57 (29.4) | 93 (100) | 8 (100) | 159 (45.6) |
| Kivalliq | 1 (1.8) | 137 (70.6) | 0 | 0 | 138 (39.5) |
| Kitikmeot | 52 (96.3) | 0 | 0 | 0 | 52 (14.9) |

CHEO Children’s Hospital of Eastern Ontario; MCH Montreal Children’s hospital; SCH Stollery Children’s Hospital; WCH Winnipeg Children’s Hospital

Cases from the Qikiqtaaluk region were identified based on a forward sortation area (FSA) of X0A; cases from the Kivalliq region were identified based on an FSA of X0C; cases from the Kitikmeot region were identified based on an FSA of X0B.
