# Supplementary material for: Urgent air transfers for acute respiratory infections among children from Northern Canada, 2005–2014
Source: PLoS One. 2022 Jul 28;17(7):e0272154. doi: 10.1371/journal.pone.0272154 (PMC9333212; doi:10.1371/journal.pone.0272154)
Supplement: S1 Data — (DOCX) [file pone.0272154.s006.docx]

# S6 Data. ICD-10 Diagnostic codes related to ARI

**A15–A19 Tuberculosis**

o A15 Respiratory tuberculosis, bacteriologically and histologically confirmed

o A16 Respiratory tuberculosis, not confirmed bacteriologically or histologically

o A19 Miliary tuberculosis

**A30–A49 Other bacterial diseases**

o A21.2 Pulmonary tularaemia

o A22.1 Pulmonary anthrax

o A31.0 Pulmonary mycobacterial infection

o A36.0 Pharyngeal diphtheria

o A36.1 Nasopharyngeal diphtheria

o A36.0 Laryngeal diphtheria

o A37 Whooping cough

o A42.0 Pulmonary actinomycosis

o A43.0 Pulmonary nocardiosis

o A49.3 Mycoplasma infection, unspecified

**A80–B34 Viral infections**

o B01.2 Varicella pneumonia

o B20.6 HIV disease resulting in Pneumocystis pneumonia

o B25.0 Cytomegaloviral pneumonitis

o B34.2 Coronavirus infection, unspecified site

**B35-B49 Mycoses**

o B37.1 Pulmonary candidiasis

o B39 Histoplasmosis

o B40 Blastomycosis

o B44.0 Invasive pulmonary aspergillosis

o B44.1 Other pulmonary aspergillosis

o B45.0 Pulmonary cryptococcosis

o B46.0 Pulmonary mucormycosis

**B50-B64 Protozoal diseases**

o B58.3 Pulmonary toxoplasmosis

o B59 Pneumocystosis

o B960 Mycoplasma pneumoniae as the cause of diseases classified to other chapters

o B974 Respiratory syncytial virus as the cause of diseases classified to other chapters

o B97.2 Coronavirus as the cause of diseases classified to other chapters

o B97.8 Other viral agents as the cause of diseases classified to other chapters

**J00–J06 Acute upper respiratory infections**

o J00 Acute nasopharyngitis (common cold)

o J01 Acute sinusitis

o J02 Acute pharyngitis

o J03 Acute tonsillitis

o J04 Acute laryngitis and tracheitis

o J05 Acute obstructive laryngitis (croup) and epiglottitis

o J06 Acute upper respiratory infections of multiple and unspecified sites

**J09–J18 Influenza and Pneumonia**

o J09 Influenza due to identified avian influenza virus

o J10 Influenza due to identified influenza virus

o J11 Influenza, virus not identified

o J120 Adenoviral pneumonia

o J13 Pneumonia due to Streptococcus pneumoniae

o J14 Pneumonia due to Haemophilus influenzae

o J15 Bacterial pneumonia, not elsewhere classified

o J16 Pneumonia due to other infectious organisms, not elsewhere classified

o J17 Pneumonia in disease classified elsewhere

o J18 Pneumonia, organism unspecified

**J20–J22 Other acute lower respiratory infections**

o J20 Acute bronchitis

o J21 Acute bronchiolitis

o J22 Unspecified acute lower respiratory infection

**J30–J39 Other diseases of upper respiratory tract**

o J36 Peritonsillar abscess

o J390 Retropharyngeal and parapharyngeal abscess

**J840–J849 Other interstitial pulmonary diseases**

o J848 Other specified interstitial pulmonary diseases

o J849 Interstitial pulmonary disease, unspecified

**J85–J86 Suppurative and necrotic conditions of lower respiratory tract**

o J85 Abscess of lung and mediastinum

o J86 Pyothorax, Empyema

**J90–J94 Other diseases of pleura**

o J90 Pleural effusion, not elsewhere classified, Pleurisy with effusion

o J91 Pleural effusion in conditions classified elsewhere

**P20–P29 Respiratory and cardiovascular disorders specific to the perinatal period**

o P23 Congenital pneumonia

o P284 Other apnoea of newborn
